# Abnormal gray matter volume and structural covariance network of basal ganglia-limbic system in patients with major depression disorder

**DOI:** 10.3389/fneur.2025.1712229

**Published:** 2025-11-20

**Authors:** Xiaoning Shao, Ruiping Zheng, Shaoqiang Han, Yuan Chen, Yong Zhang

**Affiliations:** Department of Magnetic Resonance Imaging, The First Affiliated Hospital of Zhengzhou University, Zhengzhou, China

**Keywords:** major depression disorder, basal ganglia, limbic system, structural MRI, structural covariance network

## Abstract

**Background:**

Major Depressive Disorder (MDD) is a highly prevalent neurological disorder, characterized by multidimensional symptoms that are associated with structural abnormalities across multiple brain networks. There remains a lack of systematic research into the core regions and brain maturational disruption underlying the symptoms of MDD. In this study, we aimed to assess aberrant gray matter volume (GMV) and structural covariance network (SCN) in patients with MDD compared to healthy controls.

**Methods:**

T1-weighted anatomical images of 159 patients with MDD and 121 matched healthy controls were acquired. 17-item Hamilton Depression Rating Scale (HAMD-17) was utilized to assess the clinical symptoms of MDD. Voxel-based morphometry was utilized to assess the core aberrancies of GMV in patients with MDD; a novel Gaussian kernel-based density estimation was employed to construct the individual-based SCN, network-based statistic was applied to investigate the interregional structural coordinated changes; Pearson’s correlation was applied to assess the association between these abnormalities and clinical severity in MDD.

**Results:**

Patients with MDD showed increased GMV mainly in the basal ganglia (putamen), limbic system (parahippocampal gyrus, amygdala), inferior temporal gyrus and olfactory, while decreased GMV in the diencephalic nuclei (thalamus) and precentral gyrus. SCN analyses reveal an abnormal network centered on the pallidum and hippocampus as core nodes, which encompasses three functional subnetworks: the emotional regulation network, sensorimotor network, and cognitive control network. Moreover, the decreased GMV in the thalamus and increased structural coordination between the pallidum and the parahippocampal gyrus is significantly correlated with patients’ HAMD-17 scores.

**Conclusion:**

Our findings suggest the pathophysiology of MDD may primarily lie in the abnormal morphology and interregional coordinated development of the basal ganglia-limbic system. The current results provided novel supplementary evidence for the hypothesis of structural aberrations in MDD.

## Introduction

Major depressive disorder (MDD) is a highly prevalent, recurrent psychiatric condition that imposes profound burdens on individual well-being, undermining quality of life, work productivity, and mental health and strains global public health systems (1). Clinically, MDD presents with multidimensional symptoms including anhedonia, persistent low mood, and cognitive disruptions (2, 3). The suggested mechanism of MDD is the dysfunction in emotion regulation-related systems. Accumulating evidence shows it involves a wide range of regional and network abnormalities. Thus, it is essential to clarify the core regional structural damages and interregional structural covariance anomalies and how they are associated with MDD clinical severity. Existing studies have focused on exploring the neuroimaging biomarker and brain mechanism of MDD using magnetic resonance imaging (MRI).

Over the past decades, two key lines of MRI research focused on gray matter structural alterations and structural covariance networks (SCN) have advanced our understanding of MDD’s neurobiology (4, 5). For example, by using voxel-based morphometry analysis, Cai et al. found patients with unipolar depressive disorder exhibit reduced gray matter volumes (GMV) in the right inferior frontal gyrus compared to healthy controls (HCs) (6). However, Emre Bora and colleagues argue that GMV reduction in the rostral anterior cortex was the most consistent finding of MDD (7). Moreover, our previous meta-analysis, which included 19 studies with 22 datasets, suggests that the structural abnormalities in the fronto-striatal-limbic and fronto-parietal networks are essential characteristics in first-episode MDD patients (8). These studies confirm the significant role of structural brain damage in MDD, and the structural abnormalities involve multiple brain regions and networks. Notably, the inconsistent or ambiguous findings may be attributed to the heterogeneity of MDD, as well as methodological variability or uniformity. Evidence from network analyses supports the aforementioned claims; however, group-based construction of structural covariance networks often fails to adequately represent individual cases. For instance, Han et al. reveal decreased nucleus accumbens-related network in MDD (9). Subsequently, they further elaborated on the significant differences in SCN analyses between group-based and individual-based construction of covariance networks, and emphasized the necessity of constructing individual-based SCNs (10). These results underscore the imperative of combining regional and individualized SCN analyses to elucidate the pathophysiological basis of MDD.

To address this issue, we employed VBM, a mature regional GMV assessment (11) and developed a novel, individual-based method for SCN construction. VBM analyses provide an effective method to investigate the voxel-wise GMV changes, which have been widely used in many psychiatric disorders, such as social anxiety disorder (12), schizophrenia (13) and MDD (7). Structural covariance describes the coordinated regional volumes between brain regions reflecting their common development/maturation trajectories (14). Structural covariance among brain regions is hypothesized to be related to anatomical connectivity (15) and can be influenced by mutual brain-derived neurotrophic factor (16) and activity-dependent structural plasticity (17). Given the limitation that group-level structural networks fail to represent patients with schizophrenia, Liu et al. proposed an individualized SCN analysis approach, which tries to construct the network based on the GMV features of regions in individuals (18). In line with this theory (19), we constructed individual-based SCNs in two steps: (1) using Gaussian kernel-based density estimation to extract the GM probability density sequences of each region in each subject; (2) using Pearson correlation to construct individual SCNs. Collectively, this integrated research framework is well-suited for investigating the neuropathology of MDD and will facilitate the discovery of neuroimaging substrates underlying the symptoms of the disorder.

In the current study, we aimed to achieve two primary goals: (1) characterize abnormal GMV across multiple brain regions and disruptions of SCN in MDD patients, compared to demographically matched HCs; and (2) investigate how these structural abnormalities are associated with 17-item Hamilton Depression Rating Scale (HAMD-17) scores in MDD. VBM was utilized to detect regional GMV anomalies, Gaussian kernel-based density estimation was developed to construct individual SCNs, and network-based statistic was applied to identify abnormal core SCN nodes/edges, and Pearson’s correlation was used to assess the association between structural abnormalities and clinical symptoms. We hypothesized three key outcomes: (1) MDD patients would exhibit GMV abnormalities concentrated in the basal ganglia and limbic system; (2) patients would show disrupted SCNs centered on some key regions; and (3) these morphological abnormalities of these regions and interregional coordination abnormalities underlie the severity of MDD.

## Materials and methods

### Participants and clinical assessment

A total of 159 first-episode untreated patients with MDD and 121 demographically matched HCs were enrolled in this study. MDD patients were recruited from the outpatient services of the Department of Psychiatry, the First Affiliated Hospital of Zhengzhou University, Zhengzhou, China. The diagnosis of MDD was conducted by one chief physician and one well-trained psychiatrist in accordance with the Diagnostic and Statistical Manual of Mental Disorders, Fourth Edition (DSM-IV) criteria for MDD, and all patients were in the depressed phase at the time of enrollment. HCs were recruited from the local community through poster advertisements, and all HCs were Han Chinese and right-handed. Exclusion criteria for MDD patients included: (1) comorbidity with other mental (psychotic) disorders; (2) a history of manic symptoms. For all participants (both MDD patients and HCs), additional exclusion criteria were applied: (1) use of drugs such as anesthetics, hypnotics, or analgesics in the past 1 month; (2) substance abuse; (3) a history of brain tumor, head trauma, brain surgery, or other organic diseases; (4) suffering from cardiovascular diseases, diabetes, or hypertension; (5) contraindications for magnetic resonance imaging (MRI) scanning; (6) other structural brain abnormalities revealed by preliminary MRI scan. HCs were further excluded if they had a history of serious medical or neuropsychiatric illness, or a family history of major psychiatric or neurological illness in their first-degree relatives. There were no significant between-group differences in age, gender distribution, or years of education, and information on illness duration and age of onset for MDD patients is detailed in Table 1.

**Table 1:** Demographic and clinical characteristics of MDD and HC groups.

Demographics mean (SD)	HC *N* = 121	MDD *N* = 159	Statistical value	*p-*value
Age (y)	19.26 (5.65)	18.58(4.71)	T = 1.906	0.269^a^
Gender (male/female)	60/61	83/76	*χ* = 0.188	0.665^b^
Education (y)	9.72(1.79)	9.99(1.89)	*T* = −1.139	0.256^a^
Duration (m)	16.70(19.35)	–	–	–
Age of onset (y)	17.14(4.71)	–	–	–
HAMD-17	22.56(5.69)	–	–	–

MDD, major depression disorder; HC, healthy control; SD, standard deviation; ^a^*P*-value was obtained by two-sample *t*-test. ^b^*P*-value was obtained by χ^2^ two-tailed test; y, years; m, months.

The HAMD-17 was used to evaluate the clinical state of MDD patients in Table 1. All data collection, including MRI scanning and HAMD-17 assessment, was completed on the same day for each participant to avoid variability caused by temporal changes in clinical state or scanning conditions. The study was approved by the Research Ethical Committee of the First Affiliated Hospital of Zhengzhou University, and written informed consent was obtained from each participant before the experiment, in line with the principles of ethical research.

### MRI data acquisition

MRI scanning was performed on the 3-Tesla GE Discovery MR750 scanner (General Electric, Fairfield, Connecticut, United States) at the First Affiliated Hospital of Zhengzhou University. Using an eight-channel prototype quadrature birdcage head coil, participants received a 3D-spoiled gradient echo T1-weighted scan with the following parameters: repetition time (TR) = 8,164 ms, echo time (TE) = 3.18 ms, inversion time (IT) = 900 ms, flip angle = 7 degrees, resolution matrix = 256 × 256, slice number = 188, slice thickness = 1.0 mm, and voxel size = 1 × 1 × 1 mm^3^. All structural T1-weighted image acquisitions were completed under standardized scanning protocols to ensure data consistency.

### Voxel-based morphometry analysis

Preprocessing and statistical analysis of structural T1-weighted images were performed using the VBM8 toolbox within Statistical Parametric Mapping 8 (SPM8)^1^ for investigating regional GMV alterations in neuroimaging research, consistent with methods employed in prior studies (20, 21). First, each participant’s anatomical images were subjected to uniform non-linear transformation to align with the Montreal Neurological Institute (MNI) standard template, a step that minimizes inter-subject spatial variability to ensure consistency across the cohort, and subsequent resampling to a voxel size of 1.5 × 1.5 × 1.5 mm^3^ to standardize spatial resolution for group-level comparisons. The normalized images were then segmented into three distinct tissue types gray matter, white matter, and cerebrospinal fluid to isolate gray matter by leveraging signal intensity differences between tissue classes, as done in previous structural neuroimaging analyses (10, 21). Finally, to reduce random noise and improve the signal-to-noise ratio, the segmented gray matter data were spatially smoothed using a Gaussian kernel with a full-width at half-maximum of 6 mm, a smoothing parameter selected to balance preservation of meaningful regional structural differences and minimization of artifact-driven variability, in line with established protocols from comparable studies.

### Constructing the SCN

**Figure 1:**
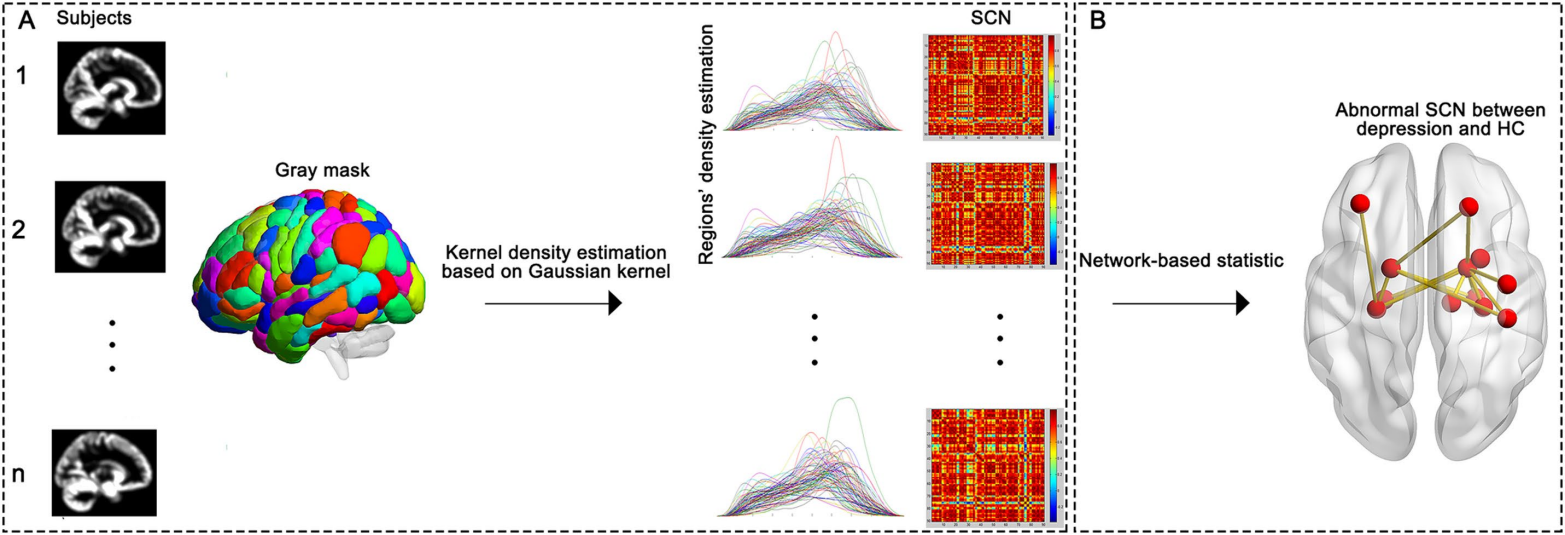
Construction of the individual-based SCN. **(A)** Illustrates the process of the construction of SCN. **(B)** Illustrates the network-based statistic, SCN, structural Covariance Network; MDD, major depression disorder; HC, healthy control.

To construct individualized SCNs for each participant (Figure 1A), we first delineated a set of gray matter regions of interest (ROIs) using the Automated Anatomical Labeling (AAL) atlas (consistent with the gray matter segmentation result from VBM preprocessing), which parcellates the whole brain into 90 functionally and anatomically homogeneous ROIs (serving as SCN nodes) (22). For each participant, Gaussian kernel-based density estimation was then applied to each ROI to quantify the probability density distribution (PDF) of gray matter within the region, with the kernel bandwidth automatically optimized to balance preservation of regional structural details and suppression of imaging noise; the resulting PDF of each ROI was further converted into a continuous gray matter probability density sequence that captures spatial distribution characteristics of gray matter in the ROI (23, 24). We then calculated pairwise interregional structural covariance to measure coordination between ROIs: specifically, we used symmetric Kullback–Leibler (KL) divergence to evaluate the statistical dependence between the kernel density estimation (KDE)-derived density sequences of each ROI pair, and transformed this divergence value into a normalized similarity score (ranging from 0 to 1, with higher scores indicating stronger structural covariance). Finally, these pairwise similarity scores were organized into a symmetric connectivity matrix (90 × 90, corresponding to 90 ROIs) for each participant, which directly constitutes the individualized SCN.

### Statistical analyses

Statistical analyses were performed using SPSS (version 25.0; SPSS Inc., Chicago, IL, United States) for clinical baseline data and MATLAB R2024b for imaging data. Descriptive statistics were utilized to summarize the baseline characteristics of the depression and HC groups, and two-tailed χ^2^ tests and two-sample *t*-tests were conducted to compare differences between groups. For voxel-wise between-group analyses, two-sample *t*-tests were applied to identify abnormal differences between patients with MDD and HCs with age, gender, and total intracranial volume (TIV) included as covariates to control for potential demographic and global brain volume confounder. Results corrected via Gaussian Random Field (GRF) correction using thresholds of voxel-level *p* < 0.001, cluster-level *p* < 0.05, and cluster size >200 voxels. To identify MDD-related abnormalities in SCN topology, network-based statistics (NBS) were utilized to compare SCNs between patients with MDD and HCs (Figure 1B), with age, gender and TIV included as covariates. This approach is designed to detect connected subnetworks with significant group differences in connectivity strength while controlling for multiple comparisons (25) (the threshold of the t-test was set to 3.1, with 5,000 permutations performed, and statistical significance was defined as *p* < 0.05), thereby enabling the characterization of aberrant SCN patterns specific to MDD.

Moreover, any region identified by the above contrasts was subjected to an additional ROI analysis. ROIs were defined using the statistically significant clusters/edges. Subsequently, Pearson’s correlation analysis was utilized between the altered GMV in the ROIs or interregional coordination and HAMD-17 scores in MDD, with age, gender and TIV included as covariates, where *p* < 0.05 was considered statistically significant.

## Results

### Demographics and clinical symptoms

Detailed demographic and clinical data for the participants are listed in Table 1. MDD and HC groups did not differ in gender, age, and years of education.

### Abnormal GMV in MDD compared with HCs

**Figure 2:**
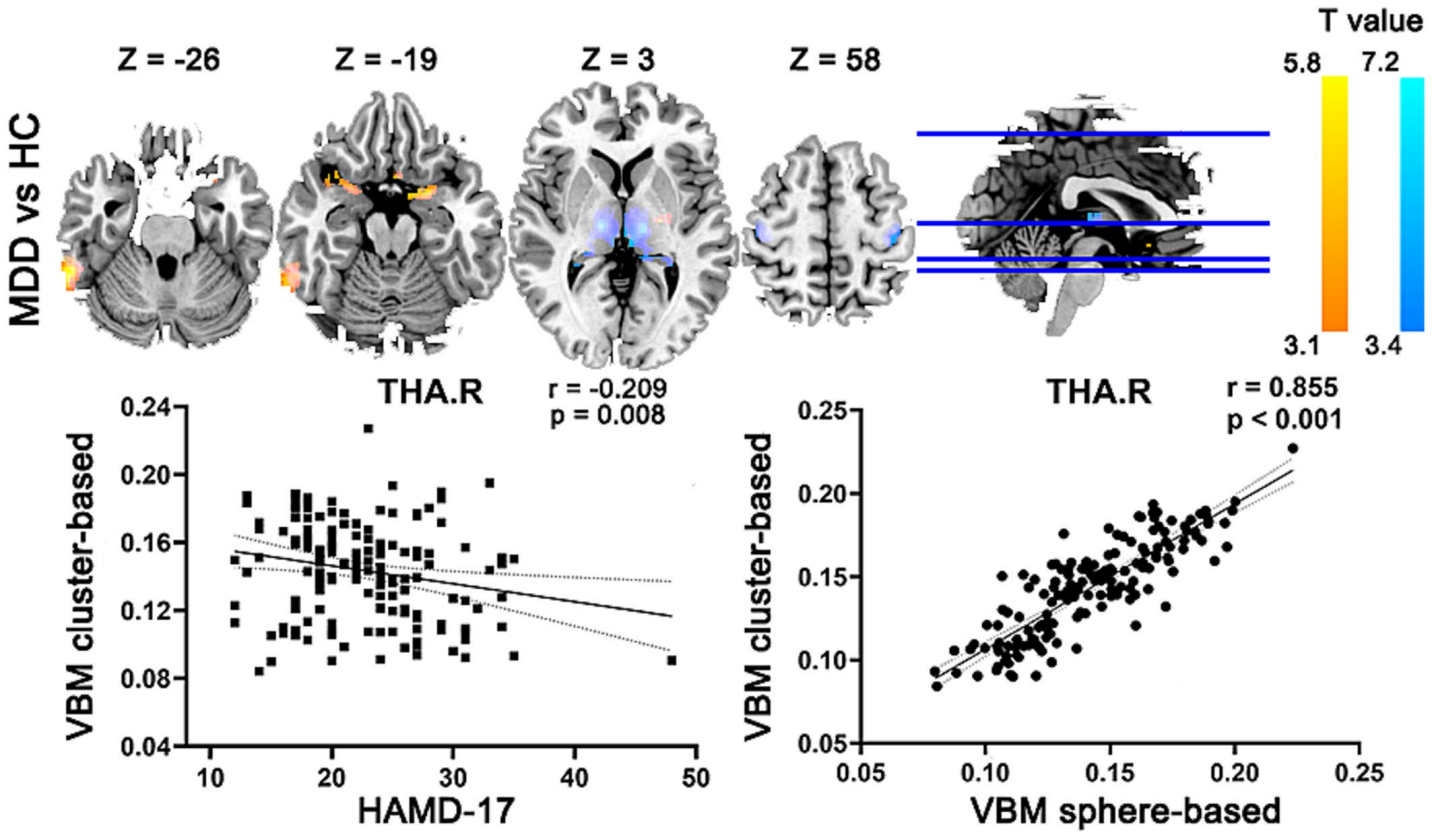
Abnormal GMV in MDD compared with HCs. MDD, major depression disorder; HC, healthy control; L, left; R, right; GMV, gray matter volume; HAMD-17, 17-item Hamilton Depression Rating Scale.

VBM analysis revealed region-specific abnormal GMV in the patients with MDD, compared with the HC group (Table 2). Patients showed significantly increased GMV mainly in the right inferior temporal gyrus, left putamen, left parahippocampal gyrus, right amygdala and left olfactory. Meanwhile, patients with MDD exhibited significantly decreased GMV mainly in the left thalamus, and bilateral precentral gyrus. Gaussian random field correction theory was employed to correct multiple comparisons (voxel *p* < 0.001, cluster *p* < 0.05, cluster size >200 voxels) (as shown in the lower left part of Figure 2). Moreover, the decreased GMV in the right thalamus are significantly inversely correlated (*r* = −0.209, *p* = 0.008, uncorrected) with the HAMD-17 in the MDD group. Meanwhile, to test the differences between signal extraction based on differential clusters and that based on spheres with a radius of 8 mm, we also conducted a sensitivity analysis. The results showed that the two were significantly correlated (as shown in the lower right part of Figure 2).

**Table 2:** Abnormal GMV in MDD compared with HCs.

Comparison	Brain region	L/R	Cluster size	MNI			Peak intensity
			Voxels	X	Y	Z	*T*-value
MDD > HC	Inferior temporal gyrus	R	611	62	−47	−26	5.94
	Putamen	L	238	−23	−14	3	5.16
	Parahippocampal gyrus	L	284	−21	3	−20	4.89
	Amygdala	R	257	26	6	−17	4.97
	Olfactory	L	221	−2	14	−17	4.53
MDD < HC	Thalamus	L	4,750	−14	−12	2	−7.91
	Precentral gyrus	R	448	41	−23	56	−5.14
	Precentral gyrus	L	704	−39	−24	61	−4.67

MDD, major depression disorder; HC, healthy control; L, left; R, right; MNI, Montreal Neurological Institute coordinates.

### Abnormal SCN in MDD compared with HCs

**Figure 3:**
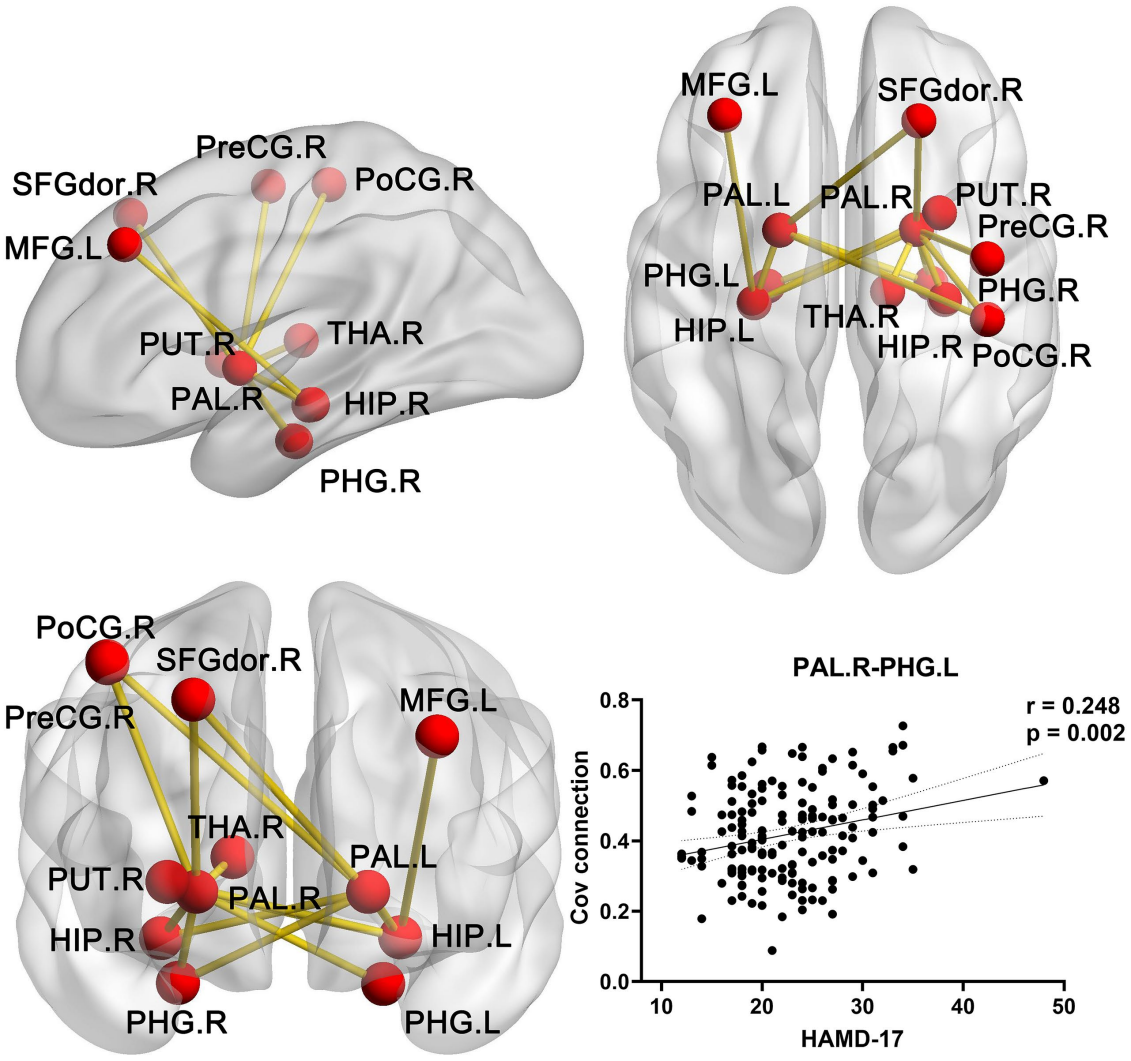
Abnormal SCN in MDD compared with HCs. Three views illustrate the nodes and edges included in the abnormal SCN. Among these, the covariant connectivity between the right pallidum and left parahippocampal gyrus was significantly positively correlated with the HAMD-17 scores in MDD. PAL, pallidum; SFGdor. R, superior middle frontal gyrus dorsolateral part; HIP, hippocampus; PHG. R, Parahippocampal gyrus; PoCG, postcentral gyrus; PreCG, precentral gyrus; THA, thalamus; PUT, putamen; Cov, covariance; L, left; R, right; HAMD-17, 17-item Hamilton Depression Rating Scale.

NBS analyses revealed an interregional structural hyper-synergistic network in patients with MDD, involving the basal ganglia nuclei (including the bilateral pallidum, right putamen), limbic system (including the bilateral hippocampus and bilateral parahippocampal gyrus), as well as regions such as the postcentral gyrus, thalamus, and middle frontal lobe in Table 3. This network was identified with a threshold of the *t*-test set 3.1, with 5,000 permutations performed, and statistical significance was defined as *p* < 0.05. Notably, the network is characterized with the pallidum and hippocampus as its core hubs (Figure 3).

**Table 3:** Abnormal SCN in patients with MDD compared with HCs.

Abnormal covariant connection	*T*-value
Left pallidum-right superior middle frontal gyrus, dorsolateral part	3.88
Left pallidum-left hippocampus	4.71
Left pallidum-right hippocampus	3.22
Left pallidum-right Parahippocampal gyrus	3.17
Left pallidum-right postcentral gyrus	3.24
Right pallidum-precentral gyrus	3.15
Right pallidum-right superior middle frontal gyrus, dorsolateral part	4.93
Right pallidum-left hippocampus	5.22
Right pallidum- right hippocampus	3.96
Right pallidum-left Parahippocampal gyrus	3.63
Right pallidum-right Parahippocampal gyrus	4.01
Right pallidum-right postcentral gyrus	3.83
Right pallidum-right thalamus	3.47
Left hippocampus-left middle frontal gyrus	3.51
Left hippocampus-right putamen	3.88

SCN, structural covariance network; MDD, major depression disorder; HC, healthy controls.

### Association between the altered structural GMV/covariance connections and clinical symptoms in MDD

The Pearson correlation analysis was performed between the altered GMV/covariance connections and HAMD-17 in MDD. In Figure 2, the GMV in the right thalamus was significantly inversely correlated with HAMD-17 in MDD. In Figure 3, the covariance connection between the right pallidum and left parahippocampal gyrus was significantly positively associated with HAMD-17 (*r* = 0.248, *p* = 0.002, FDR corrected) in MDD.

## Discussion

The current study focused on first-episode, untreated patients with MDD, combined gray structure and SCN analyses, aiming to systematically investigate abnormal GMV and aberrant inter-regional coordinated development in MDD, as well as the relationships between these structural abnormalities and the clinical symptoms. VBM analysis revealed that MDD patients exhibited increased GMV mainly in the basal ganglia, limbic system, left olfactory cortex and right inferior temporal gyrus, and decreased GMV in the left thalamus and bilateral precentral gyrus. Further, SCN-based NBS analysis identified an MDD-specific interregional structural hyper-synergistic network, centered on the bilateral pallidum (core of the basal ganglia) and bilateral hippocampus (core of the limbic system). Moreover, thalamic structural aberrations and the synergy between the basal ganglia and limbic system are significantly associated with the clinical severity of MDD. These findings collectively indicate that the pathophysiology of MDD may be rooted in morphological abnormalities of the basal ganglia-limbic system and disrupted inter-regional maturational disruption, which provides a targeted neuroanatomical basis for elucidating the pathological mechanisms underlying MDD.

### Abnormal GMV in MDD: implications for regional structural pathophysiology

The region-specific GMV abnormalities identified in MDD patients are characterised by increased GMV in the basal ganglia (e.g., left putamen) and limbic system (left parahippocampal gyrus, right amygdala) as well as decreased GMV in the left thalamus and bilateral precentral gyrus. These findings are aligned with the core functional deficits of MDD and shed light on the regional neuroanatomical basis of its pathophysiology. Our observation of increased GMV in the basal ganglia and limbic system—though seemingly contradictory to the “canonical GMV reduction” narrative—resonates with the REST-meta-MDD project (26), which also reported focal GMV increases (e.g., middle frontal gyrus) alongside decreases (e.g., middle temporal gyrus) in MDD, challenging the notion of uniform GMV loss across all disease-relevant regions. In contrast, the ENIGMA-MDD Working Group (27) prioritized subcortical volumetric analyses and identified robust hippocampal GMV reduction, particularly in patients with recurrent MDD; this discrepancy from our findings (no hippocampal GMV change) likely stems from our focus on first-episode, drug-naive patients, whereas the ENIGMA cohort included a higher proportion of recurrent cases. GMV increases in the basal ganglia and limbic system suggest adaptive or compensatory neural changes in MDD (28). The putamen, a key component of the basal ganglia that mediates the integration of motor signals and reward-related emotional valence, is thought to undergo volume increases to mitigate impaired reward sensitivity, a core deficit in MDD, exemplified by anhedonia, consistent with findings from Han et al. (10), who also reported abnormal structural covariance between basal ganglia subregions and reward-processing circuits in MDD. Similarly, the parahippocampal gyrus and amygdala, which form a central circuit for the encoding and retrieval of emotional memories, may exhibit volume elevations in response to dysregulated emotional processing; this pattern supports the notion that structural changes in these regions contribute to the persistent negative mood and rumination commonly observed in MDD patients. As validated by Serra-Blasco et al. (11) in a meta-analysis linking limbic GMV abnormalities to emotional memory dysfunction in MDD. Conversely, GMV decreases in the left thalamus, a central hub for the transmission of sensory, cognitive, and emotional signals between subcortical and cortical regions, may disrupt the coordinated transmission of information between the basal ganglia-limbic system and prefrontal cortex, thereby exacerbating emotional dysregulation and cognitive impairments in MDD. This observation aligns with Webb et al. (29), who found that thalamic GMV reduction correlates with depressive symptom severity, and Han et al., who further linked thalamic structural deficits to disruptions in the limbic-thalamo-cortical circuit (10). Furthermore, reduced GMV in the precentral gyrus, the primary motor cortex, provides a structural correlate for psychomotor retardation, a well-documented clinical symptom of MDD. This finding is consistent with a previous study that demonstrated that sensorimotor cortex GMV abnormalities in MDD are directly associated with motor symptom severity, confirming that motor dysfunction in this disorder is not solely functional but also rooted in regional structural alterations (30). Together, these GMV abnormalities reveal a distinct pattern: volume increases in regions mediating emotional processing and reward integration and volume decreases in regions supporting signal relay and motor control. This pattern highlights that MDD’s pathophysiology is not confined to isolated brain regions but involves coordinated disruptions across functionally interconnected neural systems (31, 32).

### Abnormal structural covariance network in MDD: disrupted basal ganglia-limbic coordination

The NBS analysis identified an MDD-specific interregional structural hyper-synergistic network, with core nodes concentrated in the bilateral pallidum (the core of the basal ganglia) and bilateral hippocampus (the core of the limbic system). This aligns with Long et al.’s multi-site study of 955 MDD patients, which highlighted the basal ganglia-limbic system as a core dysregulated circuit in MDD (33). It also matches Watanabe et al.’s finding of disturbed bilateral hippocampal intra-networks in first-episode drug-naïve MDD patients (34), which further confirms the limbic system’s role as a hub of structural dysfunction in MDD. Moreover, the network with abnormally elevated structural covariance in MDD can be divided into three subnetworks, including the emotional regulation network, sensorimotor integration network and cognitive control network. The abnormally increased covariance between the basal ganglia and the limbic system reflects the structural convergent changes in the emotional regulation network of patients with MDD. It is in line with the previous that basal ganglia-limbic abnormalities are trait markers of MDD (31). Moreover, we also demonstrated increased consistent structural changes in the sensorimotor integration network, including enhanced SCN between the bilateral pallidum and bilateral postcentral gyrus. These findings echo the findings of Buyukdura et al. that patients with MDD also exhibited sensorimotor symptoms (30). In the cognitive control network, hyper-synergistic connections were detected between the bilateral pallidum and dorsolateral superior middle frontal gyrus and between the left hippocampus and middle frontal gyrus. These results are consistent with a previous report by Long et al., which demonstrated that frontostriatal connectivity deficits are associated with MDD-related cognitive-emotional integration impairment (33). Collectively, these findings confirm MDD’s structural covariance abnormalities are a systemic disorder centered on disrupted basal ganglia-limbic coordination rather than scattered defects. The identified core nodes and abnormal connections serve as potential objective biomarkers, which not only reinforce the hypothesis that MDD is a network-level dysfunction (35–37) but also provide a neuroanatomical basis for targeted circuit interventions, further supplementing evidence for MDD’s structural aberration hypothesis (1).

### Clinical significance of structural abnormalities correlated with depressive severity

Correlation analyses revealed an inverse association between reduced GMV of the right thalamus and HAMD-17 scores in MDD. This finding is in line with the previous meta-analysis that smaller thalamic volume corresponded to more severe symptoms, especially anhedonia and cognitive slowing, and this is mechanistically attributed to the thalamus’s role as a relay hub in the limbic–cortical-striatal-pallidal-thalamic circuit (7, 38). Moreover, a positive correlation was identified between the enhanced structural covariance between the right pallidum and left parahippocampal gyrus and HAMD-17 scores in MDD. It reflects basal ganglia-limbic hyper-synchronization impairing emotional processing balance and worsening negative mood/rumination (39). Specifically, these correlational findings underscore that structural aberrations in the thalamus and basal ganglia-limbic coordination are not only passive markers of MDD but actively contribute to the amplification of depressive symptoms, providing a mechanistic link between regional structural changes, network-level dysfunction, and clinical severity. Moreover, this network-level association serves as a valuable marker for predicting treatment resistance (40). Notably, the right thalamic GMV and right pallidum-left parahippocampal gyrus covariance hold promise as objective biomarkers: they could not only quantify disease severity in a more precise, imaging-based manner than subjective rating scales alone but also help stratify MDD subgroups in future clinical research, addressing the long-standing challenge of MDD heterogeneity.

Some limitations of the present study should be considered. First, it lacks collection of multidimensional clinical phenotype data, only HAMD-17 were used to assess clinical status, failing to subdivide symptom subtypes or evaluate comorbid conditions, which prevents in-depth analysis of the association between structural/functional abnormalities and specific clinical symptoms Second, it is insufficient in supplementary functional neuroimaging evidence, relying only on structural or single-modal functional data without integrating other functional techniques (e.g., task-based fMRI), making it hard to fully link brain structural changes to functional impairments.

## Conclusion

This study investigated GMV and SCN abnormalities in first-episode, untreated patients with MDD. Results showed that MDD patients exhibited increased GMV in the basal ganglia, limbic system, and decreased GMV in the thalamus and precentral gyrus. SCN analysis identified an abnormal hyper-synergistic network centered on the pallidum and hippocampus, with the structural covariance between the right pallidum and left parahippocampal gyrus significantly positively correlated with HAMD-17 scores in MDD. These findings confirm that the pathophysiology of MDD primarily involves abnormal morphology of the basal ganglia-limbic system and disrupted inter-regional coordinated development, providing novel supplementary evidence for the hypothesis of structural aberrations in MDD.

## Data Availability

The raw data supporting the conclusions of this article will be made available by the authors, without undue reservation.

## Footnotes

1
http://dbm.neuro.uni-jena.de/vbm8
