# Correction: Abnormal gray matter volume and structural covariance network of basal ganglia-limbic system in patients with major depression disorder

**DOI:** 10.3389/fneur.2025.1756055

**Published:** 2025-12-09

**Authors:** Xiaoning Shao, Ruiping Zheng, Shaoqiang Han, Yuan Chen, Yong Zhang

**Affiliations:** Department of Magnetic Resonance Imaging, The First Affiliated Hospital of Zhengzhou University, Zhengzhou, China

**Keywords:** major depression disorder, basal ganglia, limbic system, structural MRI, structural covariance network

In the author list the first author was erroneously spelled “NingShao Xiao.” The correct version is “Xiaoning Shao.”

The original version of this article has been updated.

